# Journalistic Notes

**Published:** 1891-12

**Authors:** 


### Journalistic Notes.

---

The New York *Journal of Gynecology and Obstetrics* has made its appearance. It is to be issued monthly, to contain sixty-four pages, and is of standard octavo size. The editors are Dr. A. H. Buckmaster and Dr. J. D. Emmet. In addition to original communications, editorials, society proceedings, etc., the November number contains a beautiful portrait of the late Dr. Fordyce Barker as a frontispiece.

---

The *American Gynecological Journal* is the new name of the former *Journal of Gynecology.* This journal is fast making a name, and has already established itself permanently as one of the necessary special periodicals. It is edited and published by Dr.

Charles N. Smith, of Toledo, O., with the following corps of associate editors: Joseph Price, M. D., Philadelphia; Lewis S. McMurtry, M. D., Louisville, Ky.; Andrew F. Currier, M. D., New York; Walter P. Manton, M. D., Detroit, Mich.; Charles A. L. Reed, M. D., Cincinnati, O.; Henry T. Byford, M. D., Chicago, Ill.; Florian Krug, M. D., New York; William E. B. Davis, M. D., Birmingham, Ala.

